# DeepMHADTA: Prediction of Drug-Target Binding Affinity Using Multi-Head Self-Attention and Convolutional Neural Network

**DOI:** 10.3390/cimb44050155

**Published:** 2022-05-19

**Authors:** Lei Deng, Yunyun Zeng, Hui Liu, Zixuan Liu, Xuejun Liu

**Affiliations:** 1 School of Computer Science and Engineering, Central South University, Changsha 410083, China; leideng@csu.edu.cn (L.D.); zengyunyun@csu.edu.cn (Y.Z.); 2 School of Computer Science and Technology, Nanjing Tech University, Nanjing 211816, China; hliu@njtech.edu.cn; 3 School of Software, Xinjiang University, Urumqi 830046, China; xjdx_email_for_lzx@stu.xju.edu.cn

**Keywords:** binding affinity, multi-head self attention mechanism, convolutional neural network, residual network, word embedding

## Abstract

Drug-target interactions provide insight into the drug-side effects and drug repositioning. However, wet-lab biochemical experiments are time-consuming and labor-intensive, and are insufficient to meet the pressing demand for drug research and development. With the rapid advancement of deep learning, computational methods are increasingly applied to screen drug-target interactions. Many methods consider this problem as a binary classification task (binding or not), but ignore the quantitative binding affinity. In this paper, we propose a new end-to-end deep learning method called DeepMHADTA, which uses the multi-head self-attention mechanism in a deep residual network to predict drug-target binding affinity. On two benchmark datasets, our method outperformed several current state-of-the-art methods in terms of multiple performance measures, including mean square error (MSE), consistency index (CI), $r_{m}^{2}$, and PR curve area (AUPR). The results demonstrated that our method achieved better performance in predicting the drug–target binding affinity.

## 1. Introduction

The accurate prediction of drug-target binding affinity (DTA) plays an essential role in the discovery of new drugs [1], as well as drug repositioning [2,3,4]. Proteins usually act as targets to interact with small molecules to regulate important biological functions in drug discovery. Although wet-lab experimental methods have been developed to screen and characterize chemical molecules, it is time-consuming and labor-intensive to identify potential compounds on a large scale. To relieve this bottleneck, People have proposed many computational methods to identify drug-target binding affinity.

Traditional methods such as molecular docking [5,6] and molecular dynamics simulation [7] have been used in the virtual screening of compounds. Although these methods are very explanatory and even uncover the potential binding posture, their practical applications are limited, The reason is that these methods rely heavily on the existing high-quality 3D structure of the protein of interest. Besides, these methods consumes lots of computational resources.

Many methods applied machine learning to predict drug-target interactions, which was regarded as a binary classification task [8,9,10,11,12]. However, the binding affinities between drugs and targets are actually real-valued continuous variables, and some weak drug-target interactions also play important functions. So, there have been some methods proposed to predict the quantitative binding affinity representing the strength of protein-drug interactions, usually in terms of the dissociation constant ($K_{d}$), the inhibition constant ($K_{i}$), or half of the maximum inhibitory concentration (IC50). In principle, a low IC50 value (low $K_{i}$ value or high $K_{d}$ value) indicates high binding affinity. The use of continuous values to measure the binding strength is more informative. For example, Pahikkala et al. used the least-squares algorithm called KronRLS [13], which is based on the similarity of the drug-target pair calculated by Smith-Waterman (S-W) algorithm [14]. SimBoost [15] calculates drug and target ontological features and network features and then inputs them into the gradient boosting machines [16] to predict the binding affinity. CGKronRLS [17] is one of the best performers in the recent binding affinity prediction challenges of protein kinases. It uses 2D structure-based compound-compound similarity and normalized Smith-Waterman alignment scores to obtain protein-protein similarity, and then inputs the pre-calculated similarity into Kronecker kernel to calculate the binding affinity between the compounds and proteins.Pred-binding [18] method utilizes protein sequences and molecular structures combined with support vector machines [19] and random forests [20] to predict the binding affinity between proteins and compounds.

In recent years, deep learning has advanced in image processing [21], natural language processing [22], speech recognition [23], and other fields. Some studies have been inspired to develop deep learning-based methods to predict drug-target binding affinity. For example, Ozturk et al. proposed a deep convolutional neural network (CNN) method called DeepDTA [24], which uses drug SMILES [25] and sequences representation of protein as input of convolution Neural network to extract features for binding affinity prediction. WideDTA [26] used a text-based method to encode the drug SMILES and protein sequences, including four different textual pieces of information. GANsDTA [27] proposed a novel semi-supervised model based on generative adversarial networks (GANs) [28] to predict binding affinity via drug SMILES and protein sequences. DeepGS [29] is another method that takes the drug SMILES descriptors and the protein sequences to predict binding affinity. These methods have shown that deep networks can better capture the essential features than traditional machine learning algorithms. In addition to CNN, Deep-Affnity [30] combined CNN and long and short-term memory networks (LSTM) to extract sequence features, as LSTM often better captures long-distance dependency in the sequence. DeepAffnity shows that the binding affinity can be effectively predicted using only the original sequences without relying on feature engineering. Besides, some models use extended connectivity fingerprints (ECFP) [31] and graph convolutional networks [32,33,34,35] to extract drug information. However, due to the black-box nature of deep learning, the deep learning-based methods achieved remarkable performance, but these methods have limited interpretability.

In this paper, we propose an end-to-end deep learning method to predict the binding affinity of proteins and drugs. First, a multi-head attention mechanism layer is introduced to promote the associations between different features so as to identify high-order semantic features automatically. The multi-head attention mechanism can map the original features to multiple subspaces, so that our model can capture different feature associations. Second, the residual network is applied to the feature extraction layers, which allows the combination of features in a different layer. Finally, the features of the protein and drug are concatenated and fed into a fully connected layer for prediction. For performance evaluation, the Davis kinase binding affinity dataset and KIBA large-scale kinase inhibitors dataset are used to calibrate our method. We compare our method with several current state-of-the-art methods and report four performance measures, including CI, AUPR, $r_{m}^{2}$ and MSE. The experimental results show that our method is significantly better than other methods on these two datasets.

## 2. Results and Discussion

### 2.1. Evaluation Metrics

As we formulate the prediction of drug-target binding affinity as a regression problem, four metrics are used to evaluate our model: (1) the consistency index (CI); (2) the mean square error (MSE); (3) $r_{m}^{2}$ coefficient; (4) PR curve area (AUPR). The first three indicators are often used to evaluate the continuous output value of the model, and the fourth metric is used to evaluate the binary output of the model.

The Concordance Index (CI) is a model evaluation method proposed by Gönen and Heller [36]. It measures the probability of agreement between actual and predicted values. Let δi and bi denote the ground truth and predicted value of the i-th sample, respectively. Therefore, the metric is defined as follows:

(1) $$\begin{array}{c} CI=\frac{1}{Z}\sum_{\delta_{i}>\delta_{j}}b\left(b_{i}-b_{j}\right) \end{array}$$

$\delta_{i}$ and $b_{i}$ represent the measured value and predicted value of the *i*-th sample, respectively. *Z* is the normalization constant and b is the step function. The definition of $b_{\left(x\right)}$ is as follows:

(2) $$\begin{array}{c} b\left(x\right)=\left\{\begin{array}{c} 1,if\;x>0\\ 0.5,if\;i=0\\ 0,if\;x<0 \end{array}\right. \end{array}$$

This metric measures whether the predicted binding affinity values of two random drug-target pairs are predicted in the same order as their true values. The range of this value is 0–1, the closer the value is to 1, the better the model is.

The mean square error (MSE) is calculated as follows:

(3) $$\begin{array}{c} MSE=\frac{1}{n}\sum_{i=1}^{n}{\left(Y_{i}-y_{i}\right)}^{2} \end{array}$$

in which, *n* represents the total number of samples in the data set, $Y_{i}$ represents the predicted value of the *i*-th sample, and $y_{i}$ represents the true value of the *i*-th sample.

The mean regression coefficient $r_{m}^{2}$, which is proposed by previous paper [37], is calculated using the following formulation:

(4) $$\begin{array}{c} r_{m}^{2}=r^{2}*\left(1-\sqrt{r^{2}-r_{0}^{2}}\right) \end{array}$$

The area under the precision-recall curve (AUPR) evaluates the binary classification model by averaging the precision of all recall values. We choose the AUPR metric because the PR curve is more suitable than the ROC curve in the case of unbalanced data. To calculate the AUPR value, we introduce different binding affinity thresholds to convert continuous predicted values into binary values. Similar to DeepDTA, we choose threshold value 7 for Davis dataset and 12.1 for KIBA dataset.

The hyperparameters of the model are tuned through five-fold cross-validation and evaluated on an independent test set. We use the mean square error as the loss function and the Adam optimizer to minimize the loss function. Tensorflow is used to build the model and the model is trained on a workstation with two GPUs. For the Davis dataset, we use dropout and regularization to prevent overfitting. For the KIBA dataset, which is four times the size of the Davis dataset, we use only regularization to prevent overfitting.

### 2.2. Hyperparameter Analysis

For model hyperparameters, we use grid search to tune their values. The filter sizes of the three convolutional layers are set to 32, 64, and 96, respectively. For other hyperparameters, such as learning rate, batch size, and regularization, we conducted parameter tuning experiments and the final hyperparameter values are shown in Table 1. For performance evaluation on the independent test set, we run 500 epoches for training and then used for prediction.

### 2.3. Competitive Methods

To verify the superiority of our method, we compare it with six baseline methods, including both machine learning and deep learning methods. They are briefly introduced as below:

- KronRLS: KronRLS [13] is implemented based on the Kronecker Regularized Least Square, which uses Kronecker product algebraic properties to perform predictions on the whole drug-target space, without the explicit calculation of the pairwise kernels.
- SimBoost: SimBoost [15] obtains three types of features through feature engineering, and then uses gradient boosting trees trained on the extracted features to predict the binding affinity of targets and drugs.
- DeepCPI: DeepCPI [38] uses graph neural network and CNN to extract features from the SMILES of the compound and the sequence of the protein respectively. We transfer it to the regression model by modifying the neurons in the final fully connected layer to output real-value binding affinity.
- DeepDTA: DeepDTA [24] uses drug SMILES and the protein sequence as the input into three-layer CNN to learn protein and drug features, and fed into fully connected layer to predict binding affinity.
- GANsDTA: GANsDTA [27] proposes a semi-supervised generative adversarial network to predict the binding affinity of drugs and targets. This semi-supervised learning mechanism allows the method to work on unlabeled data.
- DeepGS: DeepGS [29] takes the SMILES string of the drug and the sequence information of the protein as input, and uses Prot2Vec and Smi2Vec to obtain a two-dimensional feature matrix representation of amino acids and atom. Meanwhile, graph attention network is used to extract the topological information of drugs.

### 2.4. Performance Comparison

We first conduct a performance comparison on the Davis dataset. Table 2 shows the average CI value, mean square error, AUPR and $r_{m}^{2}$ comparison. Note that DeepMHADTA1 means that only regularization is used, while DeepMHADTA2 means that both regularization and dropout are used.

It can be seen from Table 2 that DeepMHADTA2 obtained the best performance, and achieve 0.895, 0.701, 0.766 and 0.244 for CI, $r_{m}^{2}$, AUPR and MSE metric, respectively. In particular, our method performs markedly better on $r_{m}^{2}$ and AUPR. We also noticed that the traditional machine learning methods, KronRLS and SimBoost, perform slightly weak compared to deep learning-based methods. The reason lies in that traditional machine learning relies on manually curated features. As KronRLS and SimBoost use protein and drug similarity as input, while the deep learning-based methods are end-to-end learning frameworks and automatically capture the features from input data.

Next, we compared our model with other methods on another independent dataset. Table 3 shows the performance metric on the KIBA dataset. Our method achieves 0.876, 0.719, 0.806 and 0.186 of CI index, $r_{m}^{2}$, AUPR and MSE, which outperforms all competitive methods.

**Figure 1:**
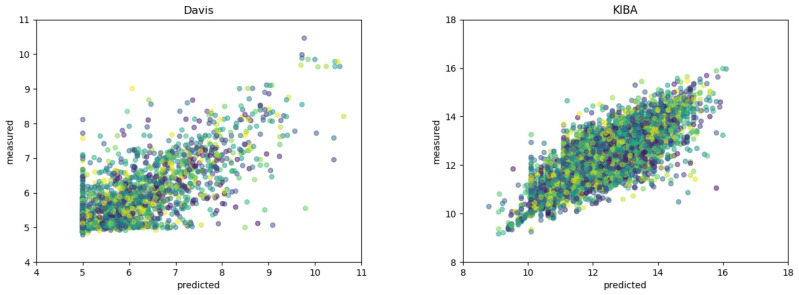
Scatter plots between the predicted and measured binding affinity values.

Moreover, Figure 1 shows the scatter plots of the measured and predicted binding affinities on two datasets. A good model should predict value as close to the true value as possible, namely, the points locate closely to the diagonal line as much as possible. It can be seen from Figure 1 that our method achieve superior performance.

To verify the effectiveness of our method, we plot the histogram of the binding affinity values in two datasets. As shown in Figure 1, binding affinity are mostly distributed in the region from 5 to 6 in the Daivs dataset, while in the KIBA dataset most values fall in the range of 10 to 15.

### 2.5. Ablation Study

The input of our model contains two parts: (1) The sequence information of proteins and drugs, and (2) the structural information of proteins and drugs. We conducted an ablation study to evaluate the impact of each part on Davis and KIBA datasets. Table 4 and Table 5 show the results. We found that on the Davis dataset, the model using only one type of information does not differ largely from the model using both sequence and structure.

However, on the KIBA dataset, the performance of the two ablation models decline significantly. The ablation study shows that the structural information of proteins and drugs plays an important role for improving model performance besides sequence information.

## 3. Materials and Methods

### 3.1. Data Source

We used two independent data sets to evaluate our model, Davis [39] and KIBA [40]. These two datasets are widely used as benchmark data sets for protein and drug binding affinity prediction. Table 6 shows the details of the two datasets.

#### 3.1.1. Davis Dataset

The Davis dataset includes the binding affinity between the kinase protein family and related inhibitors. The binding affinity value is measured by the dissociation constant $K_{d}$. This dataset contains a total of 442 unique proteins and 68 unique compounds. There are 30,056 protein-drug pairs that have binding affinity values. We use the following formula to convert the $K_{d}$ value to logarithmic form, which is adopted by previous paper [41]. The conversion formula is as follows:

(5) $$\begin{array}{c} pK_{d}=-log\left(\frac{K_{d}}{1e^{9}}\right) \end{array}$$

#### 3.1.2. KIBA Dataset

The KIBA dataset is derived from the proposed method KIBA, which combines the biological activities of kinase inhibitors from different sources (such as $K_{i}$, $K_{d}$, and IC50). The KIBA data set initially contained 467 targets and 52,498 drugs. DeepDTA refined it to include only drugs and targets with at least 10 interactions, resulting in a total of 229 unique proteins and 2111 unique drugs. The number of protein-drug pairs with known binding affinity is 118,254.

### 3.2. Model Overview

**Figure 2:**
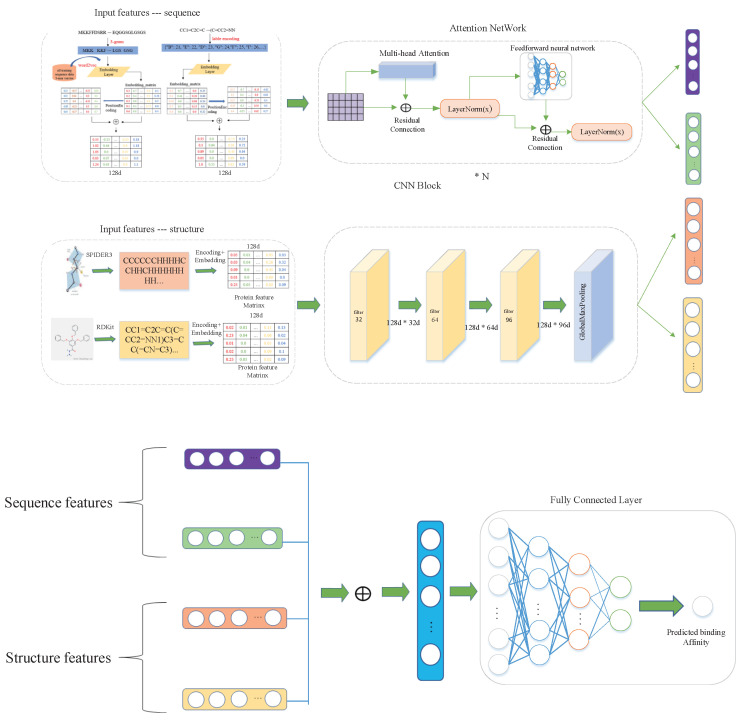
Architecture of our proposed model DeepMHADTA. The model combines sequence and structure information of protein and drug via CNN block for feature extraction, and the embeddings are concatenated as input to fully connected layer for quantitative binding affinity prediction.

We treat protein-drug binding affinity prediction as a regression problem. The overall architecture of our proposed model is shown in Figure 2. Our method automatically learns the feature of drugs and proteins to predict protein-drug binding affinity. We extracted the sequence and structural information of proteins and drugs, and combined them to feed into the fully connected layer for prediction. We obtain drug Morgan fingerprints and protein secondary structure information, by which the CNN is used to learn the latent representation (embedding). Meanwhile, we use protein sequences and drug SMILES as input into an embedding layer that projects all feature spaces and a position-coding layer that provides sequence position-coding information. The multi-head self-attention mechanism layer and residual connections are also used around each of the layers. Finally, the learned embedding are concatenated and fed into fully connected layers for regression prediction.

### 3.3. Drug Representation

#### 3.3.1. SMILES Representation of Drug

We use integer/label encoding to convert SMILES into vector. For drug SMILES descriptor, integer from 1 to 64 are used to represent a SMILES character. For example, the correspondence between SMILES string elements and corresponding integers are “@”: 8, “C”: 42, “N”: 9, “O”: 43, “=”: 40, “)”: 31, “(“: 1, “[”: 53, “Z”: 19, “]”: 54, “H”: 12, etc., the SMILES”O=C(N[C@H]” can be expressed as follows: [O=C(N[C@H] ] = [43 40 42 1 9 53 42 8 12 54].

#### 3.3.2. Fingerprint Representation of Drug

Molecular fingerprints [42,43,44] also indicate the structural features of drugs by detecting the presence of specific substructures. Integration of drug fingerprint information can provide a richer feature representation, and also allow us to study the importance of sequence and structure information in predicting drug-target binding affinity. We apply Morgan fingerprint with a radius of 2 to scan each atom of each compound, and then use a bit vector to represent the corresponding substructure. The RDKit tool is used to generate Morgan fingerprints of drugs.

### 3.4. Protein Representation

#### 3.4.1. Sequence Representation of Protein

We use the n-gram [45] method to split the protein sequence into words. In our implementation, n is set to 3. Then, the pre-trained word2vec model is used to convert the words into embeddings matrix. The word2vec algorithm [46,47] is an unsupervised model, which can encode words to low-dimensional real-valued vectors so that the words with similar semantics are also close to each other in the embedding space. We train the word2vec model to obtain a 128-dimensional embedding representation vector.

#### 3.4.2. Protein Secondary Structure

The protein [48] secondary structure is a specific conception formed by circling or folding the backbone atoms of the polypeptide chain along a certain axis, that is, the spatial arrangement of the backbone atoms of the peptide chain, and does not involve the side chains of amino acid residues. So, the protein secondary structure provides spatial structural information.

In this paper, We uses the Spider3 [49] tools which are integrated with the protein secondary structure prediction function to output the predicted protein secondary structure according to the protein sequence. For each residue in the protein sequence, SPIDER3 output three possible secondary structure states: alphahelix H, beta-strand E, and coil C. The resulting features contain important information about the close and distant interactions among amino acids.

**Figure 3:**
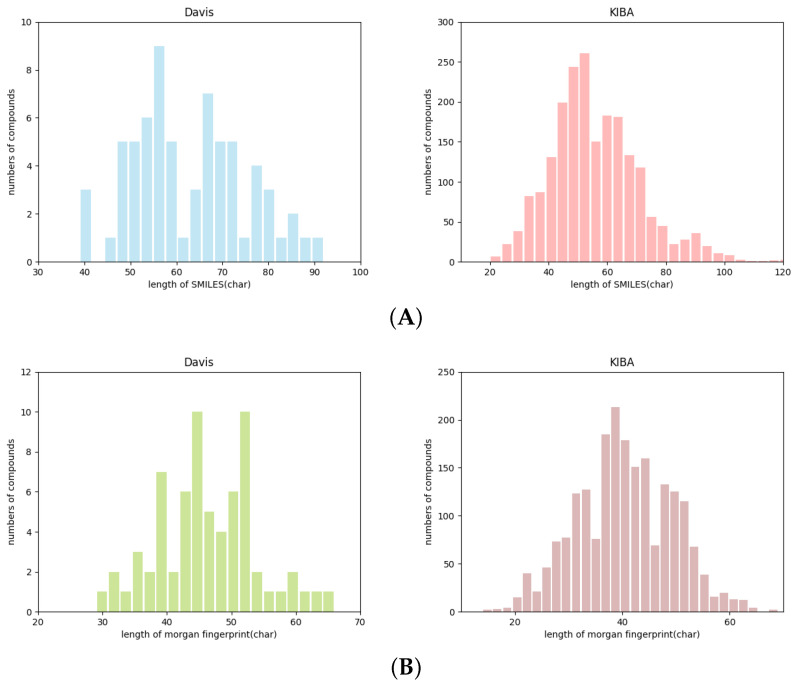

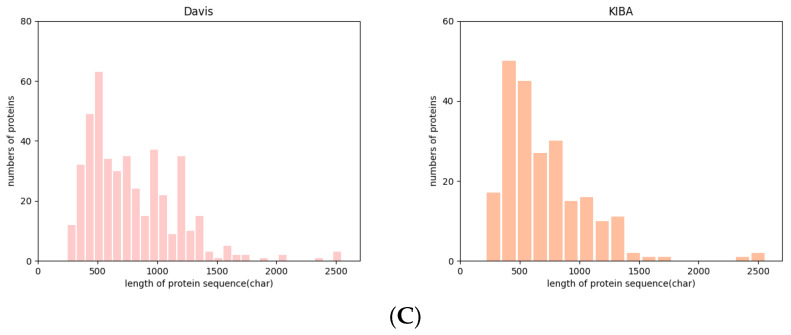
Data length distribution of Davis and KIBA data sets. (**A**) Represents the length distribution of SMILES. (**B**) The number in the middle represents the length distribution of Morgan fingerprints, and (**C**) represents the length of the distributed protein sequence.

Since the length of protein sequences, drug SMILES and fingerprints are variable, in order to extract features effectively, we compute their length distributions of proteins and drugs in the Davis and KIBA dataset. As shown in Figure 3, the protein sequence lengths range from 100 to 1000 in two datasets. For the Davis data set, we set the maximum length of Morgan fingerprint to 60. For KIBA data set, the length of the protein secondary structure is set to 1000.

### 3.5. Sequence Feature Extraction

#### 3.5.1. Sequence Embedding

Let $p=\{p_{1},p_{2},\cdots ,p_{\left|M\right|}\}$ represent the protein sequence, where $p_{i}$ represents the *i*-th amino acid, and |M| represents the length of the protein sequence. Formally, the protein p is encoded as $\left[y_{1},y_{2},y_{3},\ldots ,y_{m}\right]$, where $y_{i}$ represents the d-dimensional embedding vector of the *i*-th amino acid, so $p\in R^{\left(n\times d\right)}$ is the embedding matrix of all amino acids.

Since drug molecule is represented by the SMILES sequence, let $c=\{C_{1},C_{2},\ldots ,C_{\left|N\right|}\}$ represents the compound, where $C_{i}$ represents the tag code of the *i*-th symbol, and |N| is the length of the compound. The drug c is encoded as $\left[x_{1},x_{2},x_{3},...,x_{m}\right]$, in which $x_{i}$ represents the d-dimensional embedding vector of the *i*-th label. Therefore, $c\in R^{\left(n\times d\right)}$ is a 2D matrix formed by combining the embedding vectors of all input tags.

#### 3.5.2. Positional Encoding

The dimensions of the position-coding is the same as the sequence embedding, so the two can be summed. We use the linear transformation of the sin and cos functions to provide the model position information.

(6) $$\begin{array}{c} PE\left(pos,2i\right)=sin\left(10000^{2i/d_{model}}\right) \end{array}$$

(7) $$\begin{array}{c} PE\left(pos,2i+1\right)=cos\left(10000^{2i/d_{model}}\right) \end{array}$$

in which, pos∈[0,max_sequence_length] represents the position of an element in the sequence, i∈[0,embedding_size/2] refers to the index of the element, $d_{m}odel$ refers to the embedding dimension. Each position in the positional encoding gets a combination of values of the sin and cos functions, thereby generating unique texture positional information. After obtaining the embedding matrix and the position encoding matrix, we add the two matrix element-by-element.

#### 3.5.3. Multi-Head Self-Attention Layer

To learn the high-order features, the key problem is to determine how the embedding vectors should be combined to form meaningful higher-order features. Traditionally, this is done by domain experts creating meaningful combinations based on their knowledge. Instead, we use a novel method, the multi-head self-attention mechanism [50,51] to solve this problem. Specifically, we use a key-value attention [52] mechanism to determine the importance of each feature. Take feature *m* as an example to illustrate how to identify crucial high-level features. First, we define the correlation between feature *m* and feature *k* under a specific attention head *h*:

(8) $$\begin{array}{c} \alpha_{m,k}^{\left(h\right)}=\frac{exp(\varphi^{\left(h\right)}\left(e_{m},e_{k}\right))}{\sum_{l=1}^{M}exp\left(\varphi^{\left(h\right)}\left(e_{m},e_{l}\right)\right)} \end{array}$$

(9) $$\begin{array}{c} \varphi^{\left(h\right)}\left(e_{m},e_{k}\right)=<W_{Query}^{\left(h\right)}e_{m},W_{Key}^{\left(h\right)}e_{k}> \end{array}$$

in which $\phi^{\left(h\right)}\left(\cdot \right)$ represents the attention function used to define the similarity between feature *m* and feature *k*. It can be defined as a neural network or an inner product. Due to the simplicity and effectiveness of the inner product, we adopt the inner product $\phi^{\left(h\right)}\left(\cdot \right)$. $W_{Query}^{\left(h\right)}$, $W_{Key}^{\left(h\right)}$ represent the weight matrix that maps the original space to the new space. Next, we update the feature m in the subspace h according to the following formulation:

(10) $$\begin{array}{c} f_{m}^{\left(h\right)}=\sum_{k=1}^{M}\alpha_{m,k}^{\left(h\right)}\left(W_{Value}^{\left(h\right)}e_{k}\right) \end{array}$$

**Figure 4:**
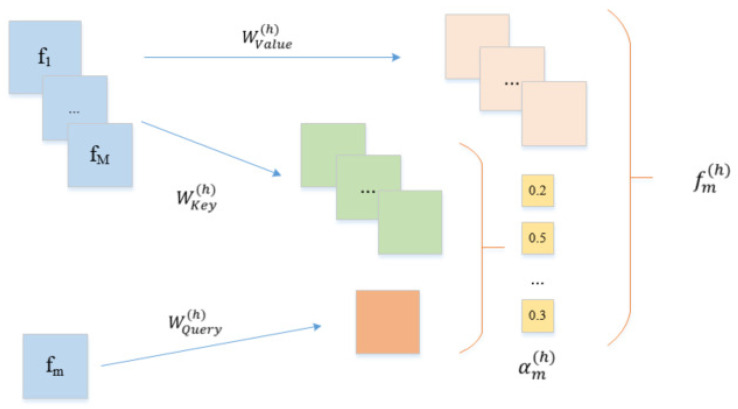
Illustrative diagram of the multi-head attention layers.

$f_{m}^{\left(h\right)}$ is the combination of feature *m* under *h* heads and related features. It represents a combination of new features learned through our method. These heads create different subspaces and learn the feature interactions of different subspaces respectively. The feature interaction process of different subspaces is shown in Figure 4. Finally, we sum the output of all different subspace features according to the following formula to obtain the final feature matrix:

(11) $$\begin{array}{c} f_{m}=f_{m}^{\left(1\right)}⊕f_{m}^{\left(2\right)}⊕f_{m}^{\left(3\right)}⊕f_{m}^{\left(4\right)}⊕\cdots ⊕f_{m}^{\left(h\right)} \end{array}$$

#### 3.5.4. Residual Connection

Residual network is a very effective network to alleviate the gradient disappearance problem and can greatly improves the depth of the network. However, many researches show that with the depth increase of the network. it is easily lead to network degradation problem, which means the performance of the network first increases and then decreases rapidly. To alleviate this problem, we use residual block [53] to connect the feature extraction layers with the original input feature like any other researches [54] according to the following formulation:

(12) $$\begin{array}{c} f_{m}^{Res}=f_{m}+x_{m} \end{array}$$

in which, $f_{m}^{Res}$, $f_{m}$, $x_{m}\in R^{dmodel\times emsize}$. The function of layer normalization is to normalize the hidden layers in the neural network to a standard normal distribution to speed up the training speed and accelerate the convergence.

## 4. Conclusions

In this paper, we propose a new end-to-end deep learning method called DeepMHADTA to predict the binding affinity of proteins and drugs. We use not only the protein sequence and SMILES descriptors of drugs, but also the protein secondary structure and drug fingerprints. For the extraction of sequence features, we used Word2Vec and label encoding to encode of proteins and drugs, respectively. Also, we combine the multi-head self-attention mechanism with the residual network as feature extraction block, and meanwhile we use the CNN to extract structural features, and finally concatenate all the embedding vectors into the fully connected layer to predict the binding affinity value. Our empirical experiments show that our method achieves superior performance on two independent datasets. We have also tried to use only sequence or structure information alone train the model, and found that both structure and sequence provide informative features. The advantage of our method is multiplex: (1) we use the multi-head self-attention mechanism, which make our model pay attention to important features. (2) For the extraction of protein sequence features, we use Word2Vec instead of label-encoding or one-hot encoding, which is a informative and efficient semantic representation than straightforward one-hot encoding. (3) We consider not only the sequence of proteins and drugs, but also their spatial structure.

## Figures and Tables

**Table 1:** Hyperparameter optimization and their tuned values.

Hyperparameters	Value
Batch_size	32
Embedding_size	128
Filter length (Protein)	12
Filter length (Drug)	4
Number of filters	[32;64;96]
num_head	8
num_block	2
Learning_rate	$1\times 10^{-5}$
Hidden neurous	[2048;1024;512;256]
epcho	500

**Table 2:** Comparison of our method with six competitive methods on Davis dataset.

Method	CI	$r_{m}^{2}$	AUPR	MSE
KronRLS	0.871	0.407	0.661	0.379
SimBoost	0.872	0.644	0.709	0.282
DeepCPI	0.867	0.607	0.705	0.293
DeepDTA	0.878	0.630	0.714	0.261
GANsDTA	0.881	0.653	0.691	0.276
DeepGS	0.882	0.686	0.763	0.252
DeepMHADTA1	0.871	0.663	0.734	0.279
DeepMHADTA2	**0.895**	**0.701**	**0.766**	**0.244**

**Table 3:** Comparison of our method with six competitve methods on KIBA dataset.

Method	CI	$r_{m}^{2}$	AUPR	MSE
KronRLS	0.782	0.342	0.635	0.411
SimBoost	0.836	0.629	0.760	0.222
DeepCPI	0.852	0.657	0.782	0.211
DeepDTA	0.863	0.673	0.788	0.194
GANsDTA	0.866	0.675	0.753	0.224
DeepGS	0.860	0.684	0.801	0.193
DeepMHADTA1	0.873	0.704	0.799	0.195
DeepMHADTA2	**0.876**	**0.719**	**0.806**	**0.186**

**Table 4:** Performance of DeepMHADTA and the ablation models using structures or sequence alone on Davis dataset.

Models	CI	$r_{m}^{2}$	AUPR	MSE
Without Structures	0.893	0.699	0.754	0.253
Without Sequence	0.853	0.588	0.698	0.358
DeepMHADTA	**0.895**	**0.701**	**0.766**	**0.244**

**Table 5:** Performance of DeepMHADTA with the Ablation model using structures or sequence alone on KIBA dataset.

Models	CI	$r_{m}^{2}$	AUPR	MSE
Without Structures	0.863	0.674	0.796	0.207
Without Sequence	0.778	0.463	0.603	0.360
DeepMHADTA	**0.876**	**0.719**	**0.806**	**0.186**

**Table 6:** Summary of the Davis and KIBA dataset.

Dataset	Proteins	Compounds	Interactions
Davis	442	68	30,056
KIBA	229	2111	118,254

## Data Availability

The data and code used in the current study is available.

## Abbreviations

The following abbreviations are used in this manuscript:

DTA	drug-target associations
CNN	convolutional neural network
RNN	recurrent neural network
MSE	mean square error
CI	consistency index
AUPR	PR curve area
SMILES	Simplified molecular input line entry specification
S-W	Smith-Waterman
LSTM	long and short-term memory networks
ECFP	extended connectivity fingerprints
PSC	protein sequence composition
